# NURSING FACILITY QUALITY, CLOSURES, AND STAFFING

**DOI:** 10.1093/geroni/igac059.1314

**Published:** 2022-12-20

**Authors:** Denise Tyler

## Abstract

The quality of care provided in nursing facilities has long been a societal concern and an important focus of policy at the federal and state level. The COVID-19 pandemic has drawn increased attention to nursing facilities due to high infection and mortality rates as well as increased media attention on staffing shortages that have occurred in the wake of the pandemic. The Office of the Assistant Secretary for Planning and Evaluation (ASPE) and RTI International have recently completed a series of studies focused on nursing facility quality and staffing. This symposium will present the results of four of those studies. First, we will report the results of a study exploring states’ use of value-based payment (VBP) programs as part of their nursing facility Medicaid payment systems. Second, we will present the results of a study examining inappropriate discharges from nursing facilities. Third, we will report findings from a study that examined the factors associated with nursing facility closures. Finally, we will present findings from a study that explored the effect of the pandemic on nursing facility staffing. Together these studies provide important information about the state of nursing facilities in the wake of the COVID-19 pandemic and suggest key areas of focus for policymakers.

